# Current Concepts in Pharmacometabolomics, Biomarker Discovery, and Precision Medicine

**DOI:** 10.3390/metabo10040129

**Published:** 2020-03-27

**Authors:** Richard D. Beger, Michael A Schmidt, Rima Kaddurah-Daouk

**Affiliations:** 1 Division of Systems Biology, National Center for Toxicological Research, U.S. Food and Drug Administration, Jefferson, AR 72079, USA; 2 Advanced Pattern Analysis and Countermeasures Group, Boulder, CO 80301, USA; mschmidtphd@patternanalysis.org; 3 Sovaris Aerospace, Boulder, CO 80301, USA; 4 Psychiatry and Behavioral Sciences, Duke Medicine and Duke Institute for Brain Sciences Duke University Medical Center, Box 3903, Durham, NC 27710, USA; rima.kaddurahdaouk@duke.edu

**Keywords:** pharmacometabolomics, pharmacometabonomics, precision medicine, drug response, metabotypes

## Abstract

Pharmacometabolomics (PMx) studies use information contained in metabolic profiles (or metabolome) to inform about how a subject will respond to drug treatment. Genome, gut microbiome, sex, nutrition, age, stress, health status, and other factors can impact the metabolic profile of an individual. Some of these factors are known to influence the individual response to pharmaceutical compounds. An individual’s metabolic profile has been referred to as his or her “metabotype.” As such, metabolomic profiles obtained prior to, during, or after drug treatment could provide insights about drug mechanism of action and variation of response to treatment. Furthermore, there are several types of PMx studies that are used to discover and inform patterns associated with varied drug responses (i.e., responders vs. non-responders; slow or fast metabolizers). The PMx efforts could simultaneously provide information related to an individual’s pharmacokinetic response during clinical trials and be used to predict patient response to drugs making pharmacometabolomic clinical research valuable for precision medicine. PMx biomarkers can also be discovered and validated during FDA clinical trials. Using biomarkers during medical development is described in US Law under the 21st Century Cures Act. Information on how to submit biomarkers to the FDA and their context of use is defined herein.

## 1. Introduction

The goal of precision medicine is to prescribe the most effective treatment to a patient with the fewest adverse effects. The reason this is challenging is that there are varying responses from patients to a drug treatment [1]. In 1998, it was reported that there were 2 million cases of adverse drug reactions (ADR) and 100,000 associated deaths in the United States per year [2]. Between the years of 1999 and 2006, the number of ADR events increased each year [3]. Simultaneously, the idea that pharmacogenetics and big data could inform personalized medicine in an effort to reduce the number of adverse effects and deliver the most effective drug to a patient was gaining popularity. Pharmacogenetics and Pharmacogenomics (PGx) are the study of how an individual’s genetic traits influence his or her response to a drug treatment [4,5,6,7]. Many genetic associations to drug metabolism and drug response have been discovered by PGx, but it’s impact on patient care has not been as great as originally expected by the pharmaceutical and regulatory communities.

As of 3 September 2019, there were 385 PGs drug label warnings for 270 FDA approved drugs or therapies [8]. PGx alone seldom predicts or explains a substantial or predominant part of intra-patient and inter-patient pharmacokinetic and pharmacodynamic variability affecting the response to a drug treatment. One major reason for limited success is that genetics does not consider contextual factors (i.e., alcohol, age, diet, polypharmacy, gut microbiota, physical activity, occupation, stress, and health), which can play a role in how a patient or group of patients respond to a drug treatment. It is now estimated that genetic factors account for only 20 to 40% of inter-individual differences in drug metabolism and response to drugs [9]. This means that, on average, 60 to 80% of the patient response is due to factors other than genetics.

Pharmacometabonomics was defined in 2006 as “the prediction of the outcome (e.g., efficacy or toxicity) of a drug or xenobiotic intervention in an individual, based on a mathematical model of *‘preintervention*’ metabolite signatures” [10]. The concepts behind pharmacometabonomics were significantly pioneered by Consortium for Metabonomic Toxicology (COMET) research at the Imperial College London in collaboration with multiple pharmaceutical companies where drugs were tested in animal models [11,12]. In contrast, pharmacometabolomics (PMx) was defined as an “enhanced understanding of mechanisms for drug or xenobiotic effect and increased ability to predict individual variation in drug response phenotypes, based on using both baseline metabolic profiles prior to treatment and also effects of drug treatment over time (‘*longitudinal*’ metabolomic profiles)” [13]. The first PMx study in 2007 evaluated a schizophrenia patient’s lipid profiles at baseline and changes from baseline to during treatment to three antipsychotics [14] that informed about treatment outcomes.

The emergence of pharmacometabolomics as a field was pioneered by the Pharmacometabolomics Research Network [15] and through a partnership with the Pharmacogenomics Research Network [16]. Seventeen academic groups worked collaboratively to provide novel insights about individual patient response variation to key drugs used to treat neuropsychiatric disorders and cardiovascular disease including antidepressants, antihypertensive statins, and antiplatelet therapies [17,18].

The network established that inclusion of metabolomics data informed about PK profiles of drugs such as statins and methylphenidate [19,20] with a role for the gut microbiota [19]; also informed about therapeutic benefit and variation among ethnic groups such as with response to betablocker atenolol [21] and also side effects of medications such as development of diabetes with the use of statins [22]. This vast body of knowledge in humans and in pharmacogenomics pharmacometabolomics studies led to the concept of including of metabolomics data could enable precision medicine [17,18].

Inherent in the definitions, both pharmacometabonomics and pharmacometabolomics research aim to study and build models that accurately predict an individual response to a drug in the clinical setting. Many reviews have already been published for pharmacometabonomics research [23,24] and pharmacometabolomics research [17,18,25,26].

Most PMx studies have focused on identifying biomarkers or metabolic patterns associated with drug metabolism, responders, non-responders, or those who experience an adverse drug response. Since samples can be collected during or after drug exposure, PMx models can be built with baseline samples or treatment samples. Treatments samples can be used to find biomarkers or mechanisms associated with drugs that do not cause a positive or negative effect for several months or years after initial dosing starts. For example, many cancer drugs have increased cardiotoxicity risk years after chemotherapy [27] and often idiosyncratic drug-induced liver injury (DILI) has prolonged latency and occurs in very few patients [28].

Often, samples collected over the course of treatment may have more relevant metabolite information (i.e., glutathione that can be lowered in oxidative stress and simultaneously produced) that can be used to predict patient response better than predictions derived from pre-dose samples. Using samples after or during dosing can lead to the discovery of provisional biomarkers that may eventually lead to their classification as pharamacodynamic response, prognostic, efficacy, safety, monitoring, and other biomarkers. This review is intended to provide a brief overview of the current strategies and future applications of clinical PMx research.

Genetics, epigenetics, sex, gut microbiota, nutrition, age, health status, occupation, and other environmental factors can impact the metabolic profile of an individual [25,26,29]. Some of these factors, including genetics, are known to influence the individual response to pharmaceutical compounds. All these factors other than genetics and sex can change in an individual over time, and during drug dosing. At the heart of pharmacometabolomics is the concept that an individual’s metabolic profile is related to the manner in which an individual responds to a drug. Metabolites that can be detected during untargeted profiling of biofluids include substrates and products of endogenous metabolism, cofactors, legal and illegal drugs, drug metabolites, dietary supplements, metabolites related to dietary intake, gut microbial metabolites, lipids, peptides, adducts, and other environmental xenobiotics. Hence, a patient’s metabolome has information about his or her current health, nutritional status, gut microbial community structure, and the mechanism or pathways affected in disease that play a role in the response to drug intervention.

The global metabolic profile of an individual can be grouped with others (such as poor or good responders to a medication based on net influences on the metabolome, or subgroups of patients who will respond similarly) and has been referred to as their “metabotype” [30]. Therefore, metabolomics profiling obtained prior to, during, or after ingestion of a drug may provide mechanistic information, biomarkers, or patterns associated with a patient’s response to that drug. Metabolic profiling related to status of energy, lipids, vitamins, gut microbial metabolites, environmental exposures, and drugs taken before, during, or after a drug response could inform clinicians about an individual patient response to drugs. If the biomarkers or mechanisms are validated in additional patient populations, the metabolic pattern could become a biomarker that could later be applied to additional studies in new patients. Initial PMx studies permit clinical discovery about metabolic signatures that could have clinical utility for understanding disease subtypes and associated patient responses.

A biomarker is defined as a characteristic that is measured as an indicator of normal biological processes, pathogenic processes, response to an exposure, or response to a therapeutic intervention. A biomarker is not simply an analyte, unless the analyte has met a set of structured criteria (FDA analytical guidance). A biomarker is also not an assessment of how an individual feels, functions, or survives. By definition, a biomarker must be analytically reproducible. Clinicians can use biomarkers to inform and to discover patterns of drug responses from individual patients. Single or a pattern of multiple biomarkers that have sufficient value and evidence supporting their use (such as those discovered during preclinical or clinical trials or after post-marketing approval) can be submitted to the FDA for biomarker qualification under the 21st Century Cures Act [31].

Leptak and co-authors described the biomarker process before the 21st Century Cures Act [32] and a new FDA guidance is being developed. Although details may change, a biomarker submission will start with a letter of intent (LOI), which details whether it is an animal model biomarker or clinical outcome biomarker and the proposed context of use (COU). The FDA can be consulted before submitting the biomarker LOI [33]. The COU is a description of how the biomarker will be used in drug development. The COU has two components: (1) the BEST biomarker category and (2) the biomarker’s intended use in drug development [34]. If the FDA accepts the LOI, a Qualification Plan (QP) can be submitted that is a detailed plan describing the necessary information that will qualify the biomarker for the proposed COU in drug development. Finally, a full qualification package (FQP), which is a comprehensive compilation of supporting evidence for the biomarker and COU, is submitted before a decision is made on the biomarker. Once the FDA approves a biomarker for a specific COU, it will be made public and can be used by anyone. Table 1 shows a list of BEST biomarker definitions and potential COU for a metabolite biomarker in each category during clinical trials.

The biomarkers and patterns of metabolites can be combined to discover pathways associated with the drug response [36,37]. Therefore, PMx research can aid in the detection of relevant analytes, improve understanding of pathways and networks, and support development of models that can be employed to predict drug responses (i.e., responders vs. non-responders). In addition, PMx can be used to discover pharmacodynamic biomarkers, which can be used to set drug dose levels or specify which drugs to prescribe for an individual. Pharmacodynamic biomarkers can also provide measures related to drug efficacy or toxicity effects. These measures, often simultaneously, provide drug pharmacokinetic (PK) information, making clinical PMx research valuable for precision medicine.

## 2. Pharmacometabolomics and “Metabotypes”

**Figure 1:**
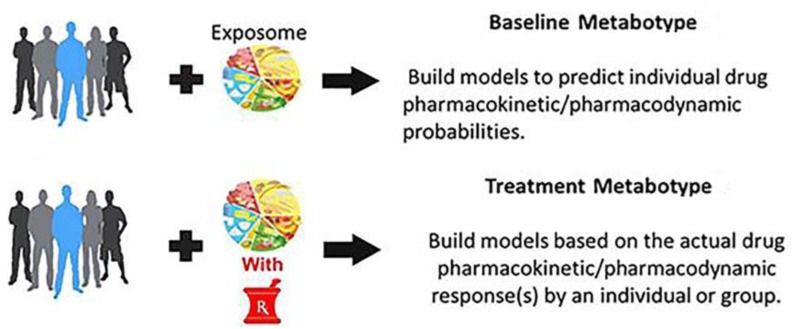
Pharmacometabolomics baseline and drug treatment approaches.

Figure 1 shows that there are two types of metabotypes in PMx studies. This includes a baseline metabotype obtained from pre-dose samples and a “treatment metabotype” derived from samples collected during dosing. Pharmacometabonomics researchers seem to prefer to limit the term to the use of baseline metabolic profiles in order to predict treatment outcomes and as parallel to pharmacogenomics [10,38]. Pharmacometabolomics researchers use both baseline metabolic profiles, as well as longitudinal drug exposure signatures to inform about response to treatment [13,18]. The resulting metabotype is a global pattern of metabolites that results from the combination of individual genetics, gut microbial genetics, gut microbial metabolites, dietary intake, and the individual’s response to his or her current exposome (nutrients, drugs, supplements, alcohol, exercise, stress, etc.). The metabolite information in the baseline and treatment metabotype can be used to discover both inter-patient and intra-patient variations in drug response. The metabolic profile, i.e., metabotype, at baseline can inform about disease subtypes and heterogeneity that arise from metabolic influences prior to treatment, such as status of the sulfur pool, environmental exposure, and nutrient status.

Baseline metabotype may play direct roles in drug responses that happen quickly, where metabolite signatures of safety or efficacy can occur quickly. The metabolic profile during treatment can be compared to baseline to determine the effect of a drug on molecular pathways and networks. This includes those pathways and networks that might be linked to adverse events. Since many drugs can cause gut microbiota or epigenetic changes before adverse events or effective response occurs, treatment samples may offer a better phenotype window for predictive PMx studies than does the baseline metabotype. Pharmacometabolomics also provides tools for mapping drug effects on metabolism and for identifying pathways that contribute to drug response phenotypes that is based on variation in response or side effects. Baseline information on metabotypes, combined with metabolite signatures for drug exposure, can potentially be used to better define mechanisms of variation in response to drug therapy and become foundations for this new field of pharmacometabolomics. Clinical PMx studies in humans have been conducted in over ten classes of drug therapies including antidepressants, statins, mood stabilizers, anti-hypertensives, and anti-platelets [22,39,40,41]. This work clearly illustrates that the genome is static, while the metabolome is dynamic. Specifically, there is rich informative data both from baseline that is obtained prior to treatment that highlights the heterogeneity of disease, and from treatment profiles that highlight individual changes in metabolism that inform and permit predictions about treatment outcome.

Drug interactions are known to occur between other drugs (polypharmacy), nutrients, dietary supplements, herbals, and other environmental exposures [42]. A drug interaction can occur during simultaneous exposure, or exposure before or after taking the drug. Drug interactions with other exposures can be classified into two primary groups: (1) pharmacokinetic (PK), which involves changes in drug absorption, distribution, metabolism, or excretion (ADME); often these changes are a direct result of interactions with phase I or phase II drug metabolizing enzymes; and (2) pharmacodynamic (PD), which are interactions that interfere with biological or physiological processes, and the changes can be additive or opposed to the drug’s primary pharmacological and toxicological effects [43]. With increases in instrument sensitivity, metabolite identification, and bioinformatics, PMx will be able to identify new drug–exposome interactions. Although most drug–exposome interactions are unknown, some drug–exposome interactions are known to cause changes in drug metabolism [44]. While the liver is the primary drug metabolizing organ, many drug–exposome interactions take place in the gut and intestine [45]. Warfarin is a drug with many known food–drug interactions that dramatically alter its efficacy and safety profile [46].

**Figure 2:**
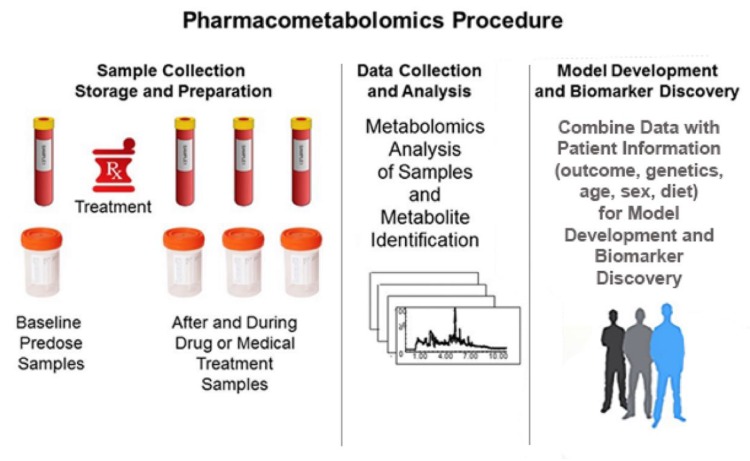
Pharmacometabolomics procedure consists of sample collection and preparation, data collection and analysis, and model development and biomarker discovery.

Figure 2 shows the typical PMx procedure. Sample collection usually consists of a baseline pre-treatment sample, followed by samples collected during and possibly after dosing. Typically, the samples collected are blood samples, but urine, feces, salvia, and breath are other types of samples that can be used. The samples are collected, stored, and prepared according to standard operating procedures (SOPs) to minimize sample degradation. Precision and attention to detail in this preanalytical stage is critical in metabolomics studies, as considerable experimental variance can be generated [47]. This experimental variance may introduce noise that scales in the context of the high variable (analyte) numbers and the resultant high dimensional data sets. The metabolomics samples are processed, and analysed with the data subsequently used to identify metabolites. The resultant metabolite data is used to build models of patient response to a drug, wherein the number of responding groups can be two or more.

It is important to use procedures to avoid false discovery of biomarkers during modelling of PMx data [48]. The discovery of biomarkers associated with the drug response can be used to discover response pathways. PMx models can be developed using baseline data, treatment data, or observed change between an individual’s baseline and treatment data. Untargeted PMx studies are discovery and hypothesis generating and need to be validated in future studies. Models can be used to determine inter-patient groups of drug response, such as slow or fast drug metabolizers or patients that are unlikely to have adverse responses. The metabolomics data obtained during treatment can be used to determine intra-patient variations in drug metabolism or drug response.

When treatment samples are used for the PMx models and it is before clinical signs of efficacy or safety are observed, the resulting potential provisional biomarkers could be considered as early predictive efficacy biomarkers for responders or early predictive safety biomarkers for patients with adverse responses. Some of the metabolites discovered in a PMx study using treatment samples may be provisional pharmacodynamic response biomarkers that could be used to set drug dose levels, such as with cholesterol in lipid lowering drugs [49]. Potentially, PMx baseline and treatment data could be collected during phase II clinical trials to discover provisional biomarkers, wherein those phase II provisional biomarkers could then be tested in phase III clinical trials.

Table 1 has a list of biomarkers types and their associated context of use during medical development. Not all biomarkers need to be discovered during the clinical trial, as biomarkers discovered in preclinical trials can be evaluated during clinical trials. Currently, clinical dose selection and PK/PD analysis is conducted during clinical trials [50]. PMx studies during clinical trials could improve dose selection and safety with the use of provisional biomarkers. Drug pharmacology involves target exposure to the drug and binding of the drug to the target, which are governed by patient genotype and phenotype characteristics [51,52]. PMx may be able to capture biomarkers associated with these pharmacokinetics/pharmacodynamic events and downstream effects of pharmacology on the pathophysiology of the patient [53]. In addition, there are off-target effects that may play roles in adverse events and overall patient response to drug. Intra-patient variations cannot be described by genetic information alone and most likely are results of genetic-environmental interactions that can change over time. Typically, at least three time points are needed to determine intra-patient variations to a drug treatment [54]. An example of drug with high intra-patient variability is tacrolimus, where high intra-patient variability (IPV), with a mean IPV of 25.1% (median = 22.6%, range: 16.2–76.0%) is an independent risk factor for adverse kidney transplant outcomes [54,55]. Another drug with high IPV (greater than 110%) is raltegravir (RAL), where the plasma level has been correlated with efficacy that was not influenced by the UGT1A1*28 polymorphism [56,57].

When a PMx study is complete, the metabolomics data should be deposited in a public metabolomics database for public use. The public metabolomics databases include MetaboLights [58], Metabolomics Workbench [59] and Consortium of METabolomics Studies (COMETS) [60]. Information about clinical metabolomics studies can be found at the Clinical Trials [61]. As of September 2019, there were 974 studies listed in Clinical Trials when searching using the term “metabolomics.” Of the 974 studies on Clinical Trials, 179 were drug intervention studies and 152 were drug supplement studies. Of the 179 drug intervention studies listed on Clinical Trials, 68 have been completed, 54 were recruiting, and 15 were active. Of the 974 studies, only 16 studies had results available and of these, 8 were in drug intervention studies. Metformin was the drug with the most studies in the database with 7 studies. Other drugs with multiple metabolomics studies were Cholecalciferol and Midazolam (4 and 5 studies, respectively).

There are four types of PMx experiments: (2.1) PMx experiments based on metabolomics data before or during drug treatment, (2.2) PMx data can be added to pharmacogenomics (PGx) data, (2.3) PMx experiments in conjunction with gut microflora genetic information (metagenomics) data, and (2.4) PMx studies where embedded medication usage, genomics, epigenomics, proteomics, metabolomics, metagenomics, and other multi-scale omics are measures within a defined clinical, environmental, or unique operational context. These are illustrated in greater detail in the following four sections.

### 2.1. PMx Data Alone

First, PMx experiments can simply be based on metabolomics data before or during drug treatment. These are like modern epidemiology experiments that are based on drug response and patient stratification [30,62,63]. The baseline metabolomics profile is used to discover biomarkers to predict drug metabolism [64,65]. The baseline metabolome is used to characterize variants within the disease cohort and then it is used to characterize the response. Finally, reference back to the baseline informs about variations in individual drug responses to discover predictive biomarkers. The metabolomics profile collected during drug treatment can be used to build models and make predictions on whether a patient (1) is a slow or fast drug metabolizer; (2) will have a favorable drug response (efficacy); or (3) will have an adverse drug response (toxicity, side effects).

The initial PMx studies used just metabolomics data and clinical responses to discover biomarkers and associated pathways related to individual drug response. The first pharmacometabonomics study found markers related to the metabolism of acetaminophen in humans and it was determined that the gut microbiota played a role in acetaminophen metabolism [10,64]. Later, several groups showed long-chain acylcarnitines were increased in blood after acetaminophen overdose in both mice and humans [66,67]. Increase in long chain acylcarnitines in plasma after acetaminophen overdose could be considered provisional safety biomarkers, since they increase before clinical signs of hepatoxicity are observed.

Early studies in humans led by the pharmacometabolomics network illustrated the promise of using metabolic profiles both at baseline and during treatment to define mechanism of action and mechanism of variation of response to treatment in ten classes of therapies. To date, PMx has been applied to study many drug classes, including antipsychotics [14,68], statins [69,70], antidepressants [19], antiplatelets [39], antihypertensives [21,71,72], cancer therapies [73,74], aspirin [75,76], metformin [72], and drug therapy side effect of treatment for amyotrophic lateral sclerosis (ALS) [77]. Novel insights were derived for each drug, illustrating that in most cases numerous biochemical pathways are impacted in a correlated way. For example, ethnic diversity in response to antihypertensives like beta-blockers was shown to have metabolic basis [21]. Sex differences in response to aspirin were highlighted, all supporting the power of information contained in a metabolic profile that goes beyond the genome.

A case for characterizing the targeted metabolome during drug therapeutics can be further illustrated by examining the drug metformin and its influence on one carbon metabolism. A series of studies has established the premise that vitamin B12 deficiency is a common sequelae of metformin administration in some populations [78,79,80,81,82,83]. Where present, the extent of the inverse effect of metformin on vitamin B12 also appears to be dose-dependent [84].

Metformin administration has also been positively correlated with elevation in homocysteine (Hcy), which is not unexpected, given the close linkage between B12 and Hcy within the one carbon metabolic network [85]. Kim et al. examined Hcy levels in 1111 patients with type 2 diabetes who took metformin for at least 6 months. Vitamin B12 deficiency was observed in 22.2% of patients. After adjusting for confounders, a 1 mg increase in daily metformin dose was associated with a 0.142 pg/mL decrease in vitamin B12. Serum Hcy levels were found to be negatively correlated with vitamin B12 levels [85].

Much of the work on metformin and Hcy is based on simple targeted analysis of Hcy. However, Orlenko et al. examined a biobank cohort exposed to metformin consisting of 546 unique adults and 42 metabolites, using automated machine learning methods. [86]. Their tandem rank-accuracy measure identified Hcy as the metabolite feature with the largest effect.

A small number of studies have examined methylmalonic acid levels following metformin administration. In the Hyperinsulinaemia: the Outcome of Its Metabolic Effects (HOME) trial, 390 insulin-treated patients with type 2 diabetes were treated with placebo or metformin for 4.3 years [78,87]. Compared to the placebo, methylmalonic acid increased with cumulative dosing (gram × years) of metformin.

These molecular dynamics also appear to be associated with the clinical phenotype. In study of 162 patients on metformin, 64% of those with diabetic neuropathy presented with low or borderline vitamin B12 levels [83]. This was compared to 17% without diabetic neuropathy. Female patients had higher levels of B12 compared to males, while those taking higher metformin doses had lower levels of vitamin B12. In the HOME trial, the increase of methylmalonic acid in metformin users was associated with significant worsening of the participant neuropathy scores [87].

These findings serve to illustrate the dynamic changes within a constrained network of metabolites in response to metformin administration and their association with a clinical phenotype. In this case, the exploratory analytes (treatment metabotype) could be considered to include vitamin B12, Hcy, and methylmalonic acid. These analytes could presently be viewed as provisional biomarkers, while research to confirm their usefulness continues. With sufficient evidence, one or more of these measures may one day achieve status as safety or pharmacodynamic response biomarkers.

Drugs for ALS have been developed to treat symptoms of the disease due to the changes in glutamate excitotoxicity, oxidative stress, energy metabolism, and amyotrophy pathways [77]. The authors discuss how metabolomics is used to identify specific metabolic pathways modified by ALS disease progression or the drug treatment, including the use of adjuvant or combined treatment to rescue these pathways. Lanznaster et al. [77] states that the combined treatments for glutamatergic overactivation, oxidative stress, and hypermetabolism are in phase I-II clinical trials. They described how PMx analysis was being used to discover clinical diagnostic and prognostic biomarkers during ALS therapy to develop better therapeutic outcomes.

### 2.2. PMx Data and PGx Data

Of great importance is the coupling of the genome and metabolome to inform about treatment outcomes. This is a concept referred to as *pharmacometabolomics informs pharmacogenomic’*, which was applied initially to the study of antidepressants [88,89] and then enlarged to the study of many drug classes [75,76,90]. Here, information from both baseline and the change in metabolism was able to benefit from and add to information derived from the genome [18,88,91]. One of the first studies to combine PMx with PGx involved discovering treatment biomarkers for citalopram/escitalopram, selective serotonin reuptake inhibitors (SSRIs) that are used to treat patients with major depressive disorders (MDD) [89]. A metabolomics assay of plasma samples from responders and non-responders taking escitalopram showed that the glycine level was negatively associated with treatment outcome (P = 0.0054) [89]. This information was used to find single-nucleotide polymorphisms (SNPs) of genes encoding glycine metabolism enzymes. It was determined that the rs10975641 SNP in the glycine dehydrogenase gene was associated with treatment outcome phenotypes (*p* = 0.02). These results highlight a possible role for glycine in escitalopram treatment for MDD.

In a follow-up study that combined PMx with genome-wide association (GWAS) studies on SSRI inhibitors, the authors determined that the use of GWAS data to identify genes in pathways identified by PMx makes it possible to rapidly accelerate PGx precision medicine studies [88]. Pharmacometabolomics and pharmacogenomics were combined to discover that purine pathway enzymes and genes were involved in the variation of patients’ response to aspirin [75]. The pharmacometabolomics and pharmacogenomics combined approach revealed that β-alanine and rs2669429 may be predictors of atenolol-induced hyperglycaemia [92]. The broad utility of the concept that pharmacometabolomics informs pharmacogenomics has been demonstrated in a series of related studies [78,81,82,83,84,85,86].

### 2.3. PMx Data and Gut Flora Metagenomics Data

A third type of PMx experiment uses gut microbiota genetic information (metagenomics) to determine biomarkers and potential mechanisms of a patient response to a drug [93]. In a landmark PMx and metagenomics study of patient’s response to an immune checkpoint inhibitor (ICT), the metagenomic analysis of fecal samples showed the gut microbiome was enriched with *Bacteroides caccae*, while PMx analysis of fecal samples revealed that anacardic acid levels were increased in all ICT responders (62-fold, *p* = 0.0077). Anacardic acid is a derivative of salicylic acid and is found in cashews and mangos [94]. Anacardic acid has been shown to supplement bactericidal activity [95]. Interestingly, patients with the highest 15:2 anacardic acid levels reported consuming cashews for several weeks before the ICT therapy. Although gut microflora levels can remain stable, diet and antibiotics can rapidly alter the gut microbiome content [96,97]. Further studies are required to validate PMx and metagenomics biomarkers for ICT responders.

### 2.4. PMx Data and Multi-Scale Omics Data

There is a fourth type of PMx study that examines embedded medication usage, while employing genomics, epigenomics, proteomics, metabolomics, metagenomics, and other multi-scale omics measures within a defined clinical, environmental, or unique operational context (e.g., military, spaceflight, occupational) [98]. One notable example of such an application is the NASA Twins study of one year in space. In this study, one male twin was on board the International Space Station (ISS) for 340 days, while the monozygotic astronaut twin served as a genetically-matched, ground control. Longitudinal assessments included the genome, epigenome, transcriptome, proteome, metabolome, microbiome, and immunome, coupled with annotation of embedded medication use [99].

## 3. Gut Microflora Metagenome and Drug Metabolism

In addition to the primary host drug metabolism system, the gut microbiota also plays important roles in metabolism of chemicals from diet, environment or xenobiotic, and pharmaceuticals through secretion of microbial active metabolizing enzymes [100]. According to the well-established evidence of gut microbial influence on pharmacokinetics, the gut microbiota usually modulates the oral drug bioavailability or half-life of drugs via microbiota-host co-metabolism by altering the capacity of drug-metabolizing enzymes or expression of genes involved in drug metabolism in host tissues.

It has been estimated that there are approximately 100 trillion cells in the human gut microbiome, which is roughly 10 times more cells than the entire human body. More recent analysis has put the ratio of human to microbial cells closer to 1:1 [101]. Although the number of cells may be the approximately the same, the gut microflora has approximately 100 times more unique genes [102,103], with the gut microbiome being arguably the most malleable of the genomes found within humans. The gut microbiota perform many functions for the host, including digestion of food components into absorbable metabolites, biosynthesis of vitamins, detoxification and removal of toxic compounds, development and regulation of the immune system, and other functions that work together between host and environment [104].

The gut microbiota can act on drugs by direct and indirect mechanisms [105]. The microbiome act directly on drugs by conversion of drugs to their active form, detoxification of drugs, and by direct binding to drugs [105]. The microbiome acts on drugs indirectly by alteration of the kinetics, production of intermediates, production of an immune response, alteration of hepatic phase I Cytochrome P450 (CYP) metabolism, and induction of enterohepatic cycling [105]. Studies with simvastatin revealed an important role for gut microbiota in the PK profile of the drug and genetic variants were identified in transporters of gut metabolites and drug metabolites [70].

There are roughly 4000 molecular entities that have been approved for human use by major markets worldwide, including the United States [106]. Despite the extensive metabolic potential of the gut microbiota, there are currently only about 40 commercial drugs that have been studied as substrates of gut microbial metabolism [107]. Yip and Chan reviewed the effect of microbiota-host co-metabolism on drug metabolism, leading to a summary of 30 drugs that are co-metabolized by host and gut microbiota [108,109]. 

It is known that acetaminophen metabolism is impacted by host-gut microbial co-metabolism [10]. Clayton et al. administered 1 gram of acetaminophen to humans, while assessing urinary *p*-cresol sulfate, acetaminophen sulfate, and acetaminophen glucuronide [64]. *p*-Cresol is derived from tyrosine and phenylalanine via gut microbial metabolism by species within the firmicutes, bacteroidetes, actinobacteria, and fusobacterium phyla [110]. Individuals with high pre-dose urinary levels of *p*-cresol sulfate had a lower post-dose urinary ratio of acetaminophen sulfate to acetaminophen glucuronide.

*p*-Cresol that is formed in the gut requires phase II sulfonation in the liver. The sulfonation process of *p*-cresol apparently competes with available hepatic sulfur groups for the metabolism (conjugation) of other substrates, such as acetaminophen. When acetaminophen is delivered to those in whom *p*-cresol is being produced by gut bacteria (e.g., Coriobacteriaceae and Clostridium clusters I, IV, IX, XI, XIII, XIVa, and XVI), there is competition for hepatic sulfur groups between the two substrates. When competition in substrate sulfonation occurs, acetaminophen metabolism is partially shunted toward glucuronidation, leading to formation of higher levels of acetaminophen glucuronide (and lower levels of acetaminophen sulfate). 

A small amount of acetaminophen is metabolized via CYP450 2E1 under normal conditions. This process can lead to the formation of the hepatotoxin *N*-acetyl-p-benzoquinone imine (NAPQI). NAPQI is detoxified by conjugation via glutathione to form a NAPQI-GSH. Also, both *p*-cresol and NAPQI are competitive substrates for sulfotransferase A1 (SULT1A1). Therefore, higher levels of *p*-cresol derived from gut microbiota may theoretically result in higher NAPQI levels. It is known that high doses of acetaminophen are toxic [111].

As a first step, normal phase 2 acetaminophen sulfonation and glucuronidation metabolism is saturated and that allows more NAPQI to be formed [111]. Second, NAPQI is known to bind to proteins, cause mitochondrial dysfunction and exhaust the liver stores of reduced glutathione (GSH) as well as the liver’s capacity to synthesize new GSH [112]. A mathematical model of glutathione depletion over time for acetaminophen toxicity has been formed and it is known that N-acetylcysteine treatment can help restore GSH levels [112].

There have been several SNPs analyzed in acetaminophen toxicity and NAPQI formation [113,114]. It is hypothesized that in patients with a genetic mutation that effects sulfur conjugation activity, the conjugation pools may need to be higher to avoid adverse events. However, studies that examine SNPs of enzymatic conjugation, coupled with the status of corresponding glutathione and sulfur pools, have not been performed.

**Figure 3:**
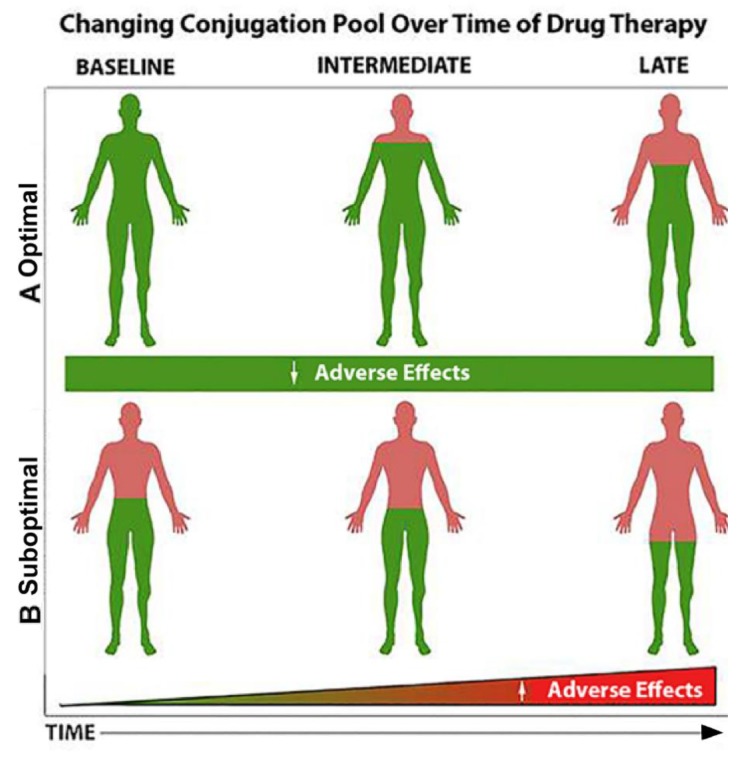
How the pool of drug-conjugating nutrients may influence the development of adverse effects with time.

Figure 3 shows a hypothetical depiction of how an enriched conjugation pool may influence the response to drug ingestion over time. (A) When the pool of conjugating nutrients (when green is at optimal levels; e.g., glutathione) is high, adverse biological effects and adverse events are expected to be lower. Similarly, when substances that are central to the conjugation pool are provided concomitant with drug use (e.g., N-acetylcysteine combined with acetaminophen), the sulfur pool is conserved, and adverse effects are minimized. (B). When the pool of conjugating nutrients (e.g., glutathione) is suboptimal (when green is depleted or severely reduced; e.g., glutathione) or no N-acetylcysteine is provided, adverse biological effects and adverse events are expected to be higher (Figure 3B). Similar scenarios could be envisioned for vitamin B12 levels as described for metformin earlier. There are many drugs that have effects on nutrients, [115] and nutrients like methionine can effect cancer and epigenetics [116].

Statins are also influenced by gut–microbiome interactions. Statins are cholesterol lowering medications that have large variability in patient response. PMx analysis identified bacterial-derived bile acids that were related to low-density lipoprotein cholesterol (LDL-C) lowering in patients that responded favorably to simvastatin. Genetic analysis of the patients identified associations between levels of several bile acids and a SNP in the gene encoding the organic anion transporter SLCO1B1 [19].

Gut microbes may also contribute to circulating non-ovarian estrogen levels with the production of β-glucuronidase. This microbial influence may warrant consideration when prescribing estrogens or anti-estrogenic drugs. Specifically, β-glucuronidase is produced by a wide spectrum of gut microbes. This enzyme deconjugates glucuronidated estrogens, liberating free fecal estrogens to be reabsorbed back into circulation. This contributes to the total circulating estrogen to an extent that warrants further clarification in vulnerable populations, for example, in the management of breast cancer.

In this regard, Flores et al. examined the fecal metagenome (using 16S rRNA amplicons), urine, and serum estrogens [117]. In men and postmenopausal women, the richness and diversity of the fecal microbiome was associated with levels of total urinary estrogens (R ≥ 0.50, *P* ≤ 0.003). These urinary estrogens were significantly associated with fecal *Clostridia* taxa, including non-*Clostridiales* and *Ruminococcaceae* (R = 0.57−0.70, *p* = 0.03−0.002). Urinary levels of Estrone, but not other estrogen metabolites, was correlated with functional activity of fecal β-glucuronidase (R = 0.36, *p* = 0.04) [117].

In another example, analysis of the gut metagenome coupled with PMx was able to decipher differences in patients to digoxin-induced cardiotoxicity [100]. Digoxin has a very narrow therapeutic window, requiring careful monitoring to avoid toxicity. Digoxin inhibits Na^+^/K^+^ATPases in cardiomyocytes, causing an influx of calcium and enhanced muscular contraction. A combination of culture-based studies, sequencing, and bioinformatics identified microbial genes *E. lenta* associated with reductive metabolism leads to digoxin inactivation in humans [100].

## 4. Where We are Today and the Future of PMx

While the analytical capability of metabolomics has been rapidly improving, the clinical use of PMx has proceeded more slowly. There are several types of PMx experiments; PMx alone, PMx combined with PGx, PMx combined with gut microflora metagenomics, and PMX with multi-scale omics data. PMx is following PGx, which went through rapid growth, but was also slow to be applied in many clinical settings. The slow adoption of PGx and PMx appears to be due to a combination of ethical, monetary, medical, and organizational issues. Nonetheless, the concept behind precision medicine is that clinical pharmacology, medical records, PGx, and PMx information can be augmented with machine learning methods to aid patient care and medical decisions.

The combination of PGx and PMx information can provide the genetic, drug metabolite, systemic metabolite, and environmental information to better understand individual patient responses to drugs. Fundamentally, unpredictable drug metabolism impacts all aspects of human health and safety in medicine. Currently, clinical dose selection and PK/PD analysis is conducted during clinical trials [50]. Treatment samples are collected in clinical trials to determine PK. Adding PMx to PK analysis can provide information to further understand responders (drug efficacy), adverse events (safety response), and the metabolic signature (metabotype) of a patient’s drug response. If the drug efficacy or safety response is before clinical signs, the biomarkers can be considered a provisional predictive efficacy or provisional safety biomarker. The 21st Century Cure Act specifically states that biomarkers can be submitted during medical development. PMx can be used to discover provisional biomarkers besides predictive and safety biomarkers, such as pharmacodynamic response biomarkers, monitoring biomarkers, and biomarkers to aid patient selection in clinical trials. Information for submission of biomarkers to the FDA is provided herein.

The FDA currently accepts biomarker information, including PGx biomarkers in a submission package for a New Molecular Entity (NME) or Biologic License Application (BLA) [118]. As of 2013, 80% of PGx studies were conducted post market by academic labs and not pharmaceutical companies [119]. There are 385 genomics drug label warnings for 270 FDA approved drugs, but the genomics label warnings do not cover the majority of the intra-patient and inter-patient variability to these drugs. There is a need to understand how environmental factors impact drug response and PMx can provide information on xenobiotic, endogenous and gut-microbe metabolites that are circulating in a patient before, during, and after drug exposure. These chemical-chemical interactions may play a role in how a patient will respond to a drug treatment and can be considered PD response biomarkers.

In the last decade, PMx has shown the ability to improve predictions when combined with PGx [18,88,90,120]. The concept that “pharmacometabolomics informs pharmacogenomics” is now being tested in many clinical centers. PGx, coupled with PMx and related disciplines, can advance the pursuit of safety and clinical outcomes for patients. This advancement, however, will require strong advocates, along with formalized and organizational efforts across the medical, clinical, and regulatory landscape. The combination of PGx and PMx has been shown to make better predictions of responders and safety. Thus, blood and urine samples for PMx should be collected whenever possible during clinical drug studies. Attention to specimen integrity and pre-analytical issues will be crucial for the success of PMx and PGx, since specimen stability strongly influences experimental variance (and ultimately data integrity). It may take years to realize the full value of PMx but improving medical decisions by coupling PMx and PGx should bring substantial value to the practice of medicine.

## Figures and Tables

**Table 1:** Pharmacometabolomics Biomarkers.

BEST Biomarker Category	Relationship between Metabolites and Biomarker Category	Potential Context of Use (COU) in a Clinical Study
Prognosis Biomarker	Metabolites that indicate a likelihood of a future clinical event	Stratify Patients Enrichment: Inclusion/Exclusion Data
Diagnostic Biomarker	Metabolites that detect the presence of a disease or identify individuals with a subtype of the disease	Patient Selection
Monitoring Biomarker	Metabolites that are measured continually over time to assess status of a disease or medical condition or for evidence of exposure to (or effect of) a medical product or an environmental agent	Indicate Toxicity or assess safety Provide evidence of exposure
Predictive Biomarker	Metabolites that predict outcome	Identify individuals based on effect from a specific intervention or exposure
Safety Biomarker	Metabolites that are related to adverse and safety events	Indicate the presence or extent of toxicity related to an intervention or exposure
Pharmacodynamic Response Biomarker	Metabolites that are related to response in an individual or group of individuals who have been exposed to a medical product or an environmental agent	Efficacy biomarkers/surrogate endpoint Show biological response related to an intervention or exposure
Susceptibility/Risk Biomarker	Metabolites related to developing a disease or medical condition in an patient that does not currently have clinically apparent disease or medical condition	Indicate the potential for developing a disease or sensitivity to an exposure
Provisional Biomarker	Metabolites that are in discovery and show potential as biomarkers, although they have not been validated as true biomarkers	Discovery-associated analytes that assist in identification of signals with potential biological meaning.

*BEST (Biomarkers, EndpointS, and other Tools) Resource [35].
